# Application of venous flow-through free flap for multi-finger revascularization and soft tissue defect coverage: a mid-term follow-up

**DOI:** 10.1186/s12893-025-03251-7

**Published:** 2025-10-27

**Authors:** Qinci Xie, Jinying Wang, Lin Huang, Junjie Zheng, Shiqiang Zhuo, Xiaofeng Chen, Yongfeng Li, Jiezhi Dai, Gaofeng Huang

**Affiliations:** 1 Department of Orthopedic Surgery, Jinjiang Municipal Hospital, Shanghai Sixth People’s Hospital Fujian, Jinjiang, Fujian China; 2 Department of Orthopedic Surgery, Shanghai sixth people’s hospital, Shanghai Jiao Tong University School of Medicine, Shanghai, China

**Keywords:** Venous flow-through flap, Free flap, Multi-finger injuries, Microsurgery

## Abstract

**Objective:**

To investigate the treatment outcome of applying venous flow-through free flap for multi-finger injuries with revascularization and soft tissue defect coverage.

**Methods:**

Between January 2021 and May 2025, 22 cases (51 fingers) with soft tissue defect and vascular defect were treated with venous flow-through flaps. There were 14 males and 8 females, aged 22–50 years (mean, 35years). There included 15 cases of two-finger defects and seven cases of three-finger defects. Functional evaluation were assessed including sensory recovery (S-2PD Test), range of motion (TAM score), and time to return to daily function or work.

**Results:**

Mean follow-up was 11 months (range 8 months to 16 months). The size of defects ranged from 1.2 cm ×1.6 cm to 2.0 cm × 3.2 cm, and the size of flaps ranged from 1.5 cm ×1.8 cm to 2.7 cm × 4.0 cm. Eight cases had some degree of swelling, and blistering at the margin of the flap was observed in two patients. All the flaps and donor sites healed completely. All of the flaps recovered protective sensation with the score of 2-PD 15.78 ± 1.30 mm. Using the contralateral finger as a control, the affected finger showed a mean TAM ratio of 82.55% ± 6.35%. 48 fingers were classified as good, and three fingers were classified as fair. The average time to return to daily function or work was 3.64 ± 0.90 months.

**Conclusion:**

Venous flow-through free flap can repair both vascular defect and soft tissue defect for complex multi-finger injuries. The flap is thin, flexible, with easily identifiable vascular distribution, and no arterial sacrifice, with low donor site morbidity.

Multi-finger trauma can present a significant challenge for hand surgeons. The extensive soft tissue defects with vascular injury often result in fingers amputation. Immediate revascularization with soft tissue coverage is required in this situation. However, appropriate reconstructive options are limited.

In 1981, Nakayama et al. [1] first described the concept of venous flaps in rat studies, and Honda et al. [2] reported its clinical use of a composite flap with arterial flow-through finger revascularization and soft tissue coverage. Since then, the venous flow-through free flap has been used for the hand and upper extremity reconstruction. This flap utilizes the superficial vein system as the conduits to provide inflow and outflow of the flap [3], and has the following advantages, such as simultaneous vascular and soft tissue reconstruction, ease of flap harvesting, low donor site morbidity, and no arterial sacrifice [4]. Roberts et al. [5] performed a systematic review of the literature and concluded that the use of venous flaps for concomitant revascularization and soft tissue coverage in traumatic hand injuries permitted good results with limited morbidity. Many different designs and applications for venous flaps in traumatic hand injuries have been reported. However, few studies have reported the application of venous flow-through free flaps for multi-finger revascularization and soft tissue defect coverage in a single operation [6]. In this study, we present our experience with venous flow-through free flaps for multi-finger injuriy salvage. This flap is harvested from the volar of the forearm. We believe this technique can be safely performed in a single operation with a successful outcome.

## Patients and methods

### Study design

This is a single-center, retrospective study conducted from Jan 2021 to May 2025 at the Department of Orthopedic Surgery, Jinjiang Municipal Hospital, Jinjiang City, FuJian Province, China (Shanghai Sixth People’s Hospital Fujian) (Ethics approval number: AF-07–08-1.0). Authors adhere to the STROBE guidelines.

### Patients

From Jan 2021 to May 2025, 22 patients (51 fingers) with soft tissue defect and vascular defect were treated with venous flow-through free flaps. The inclusion criteria were as follows: (1) multi-finger injuries; (2) soft tissue defect with exposed tendons, bones, or nerves, (3) bilateral digital arteries injured. The exclusion criteria were as follows: a destructive injured finger or a mangled upper limb; serious wound contamination or infection; and a complicating serious medical condition. There included 15 cases of two-finger defects and seven cases of three-finger defects. There were 14 males and 8 females, mean aged 35.0 ± 8.0 years (range, 22–50 years). The studies involving human participants were reviewed and approved by the Ethic Review Board of Jinjiang Municipal Hospital (Shanghai Sixth People’s Hospital Fujian). Informed consent was obtained from all participants involved in the study.

### Surgical technique

**Fig. 1:**
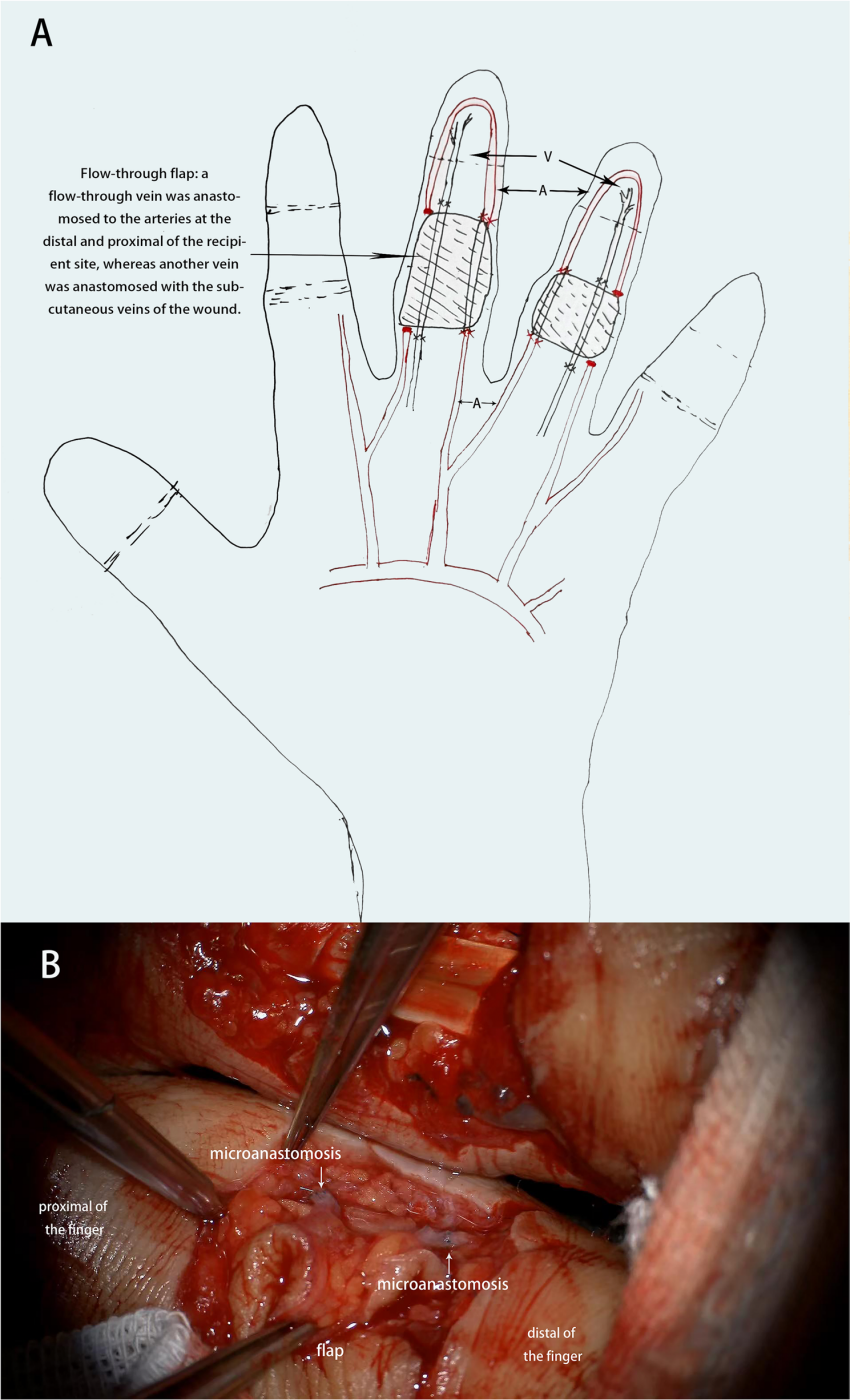
Schematic diagram (**A**) and vascular anastomosis diagram (**B**)

Techniques and designs described in this studies were performed by senior surgeons in a matured team of microsurgeons with knowledge and regular experience in this field. Brachial plexus anesthesia was administered with arm pneumatic tourniquet control before surgery. The schematic diagram and vascular anastomosis diagram were presented in Fig. 1. All the flaps were harvested from the forearm of the ipsilateral extremity. After debridement, the recipient digital artery and vein were identified and marked. A paper template with the same dimensions as defect was created. All the flaps were tailored to a size 5–8 mm larger than the paper template to alleviate possible postoperative swelling and edema. According to the wound template, two to three separate venous flow-through free flaps were designed and harvested on the volar side of the forearm. The skin along the edge of the flap was incised, and the number and positions of veins at the flap edge were observed and marked. Veins were carefully detected along the distal and proximal of the flap for approximately 5 mm and cut. Then the remaining borders of the flap were incised. After the flap was elevated and applied to cover the defect, the distal vein of the flap was anastomosed to the digital artery on the distal side of the flap, and the proximal vein of the flap was anastomosed to the digital artery on the proximal side. Usually, the vein was anastomosed to the dominant side artery of the finger. After good blood flow was confirmed by the patency test, the other vein of the flap was anastomosed to the distal and proximal end of the subcutaneous vein of the wound, respectively. Finally, the donor site defect was covered with a split thickness skin graft or closed primarily. The defect in other finger was reconstructed with the same procedure.

### Postoperative management and follow-up

Standard postoperative free flap care and monitoring were performed for one week. Skin flap color, capillary filling, and temperature were monitored every two hours for the first three days, and changed to every four hours for the following four days. The dressing is kept loose to avoid pressing on the flap and obstructing blood flow. Low-molecular-weight heparin (5000 IU per day) were used to prevent thrombosis. Vascular emergent surgery would be performed when the flap color turned gray. During this period, the survival of flap, early complications (e.g., infection, swelling, hematoma, and venous congestion) were intensively observed and recorded.

After the operation, the affected hand was asked immobilized in a functional position for approximately one weeks, and was instructed to avoid strenuous exercise for 3 weeks. Passive functional exercise was performed after two weeks, and one month later, active flexion and extension exercises would be conducted.

All patients were followed up every one month. At the final follow-up, functional evaluation, including sensory recovery and range of motion, were performed and recorded. Sensory recovery was assessed with static two-point discrimination (2-PD). Function of the finger was evaluated using the total active movement (TAM) score. It was calculated as the difference between total active flexion (MCP + PIP + DIP) and total extension deficit (MCP + PIP + DIP). The TAM score were assessed as a ratio to the contralateral finger, from here on referred to as TAM ratio. The grade was classified as excellent (100% normal), good (75–99% normal), fair (50–74%), or poor (< 50% normal). We also recorded the time to return to daily function or work.

### Statistics

All statistical analyses are performed using SPSS 18.0 Windows edition (SPSS, Inc. Chicago, IL, USA). Descriptive data were given as mean and standard deviation (SD) for quantitative variables and percent for qualitative variables.

## Results

**Fig. 2:**
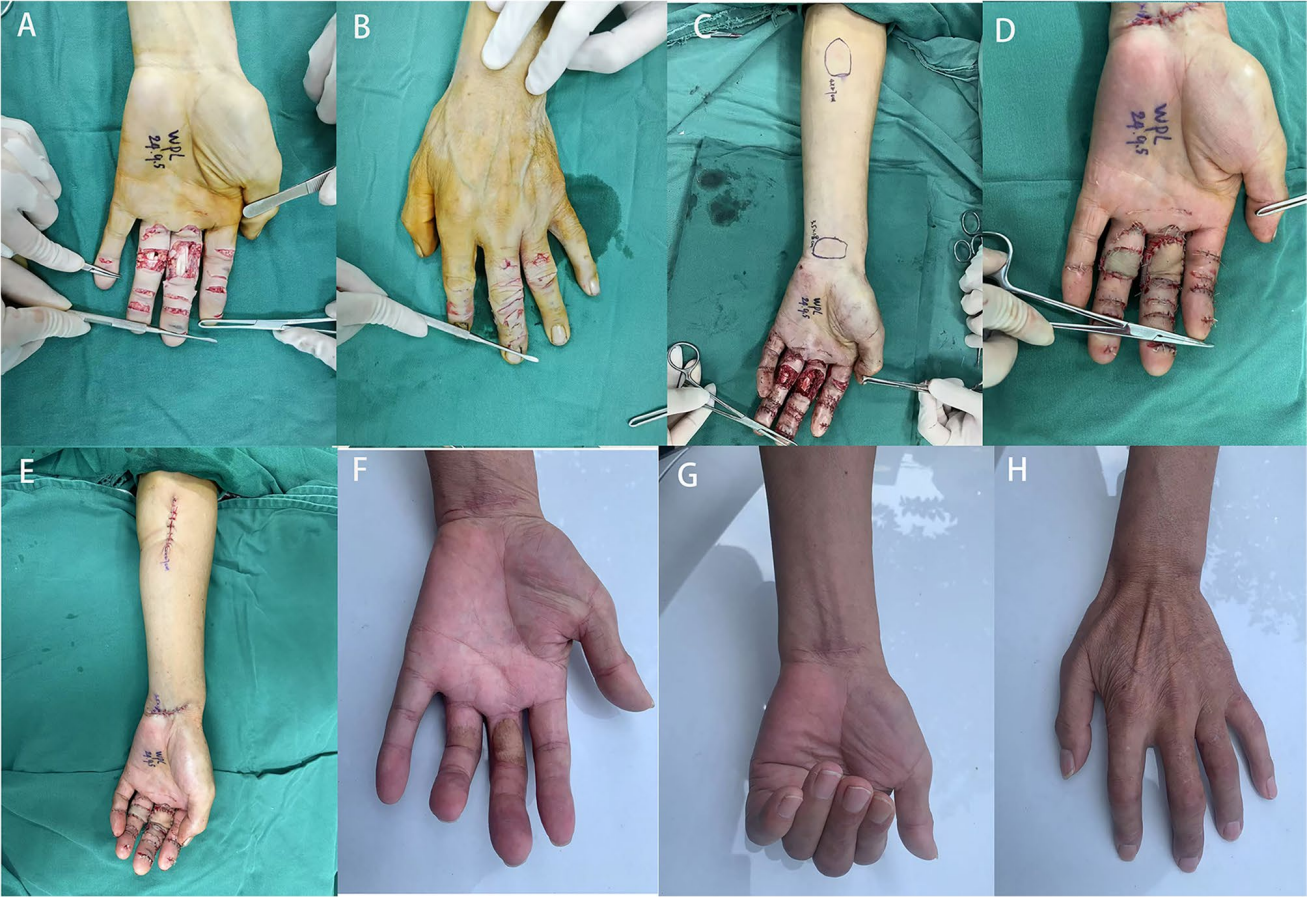
Case 1: a 48-year-old female suffered with multiple soft tissue defects and vascular injures in her left middle and ring fingers caused by a machine crush injury. **A**-**B**, Preoperative view of the injured hand. **C** Two venous flow-through free flaps were designed from the left arm. **D**-**E** Immediate postoperative view of the flaps and the donor-site. **F** Postoperative view at follow-up. **G**-**H** Postoperative functional view

**Fig. 3:**
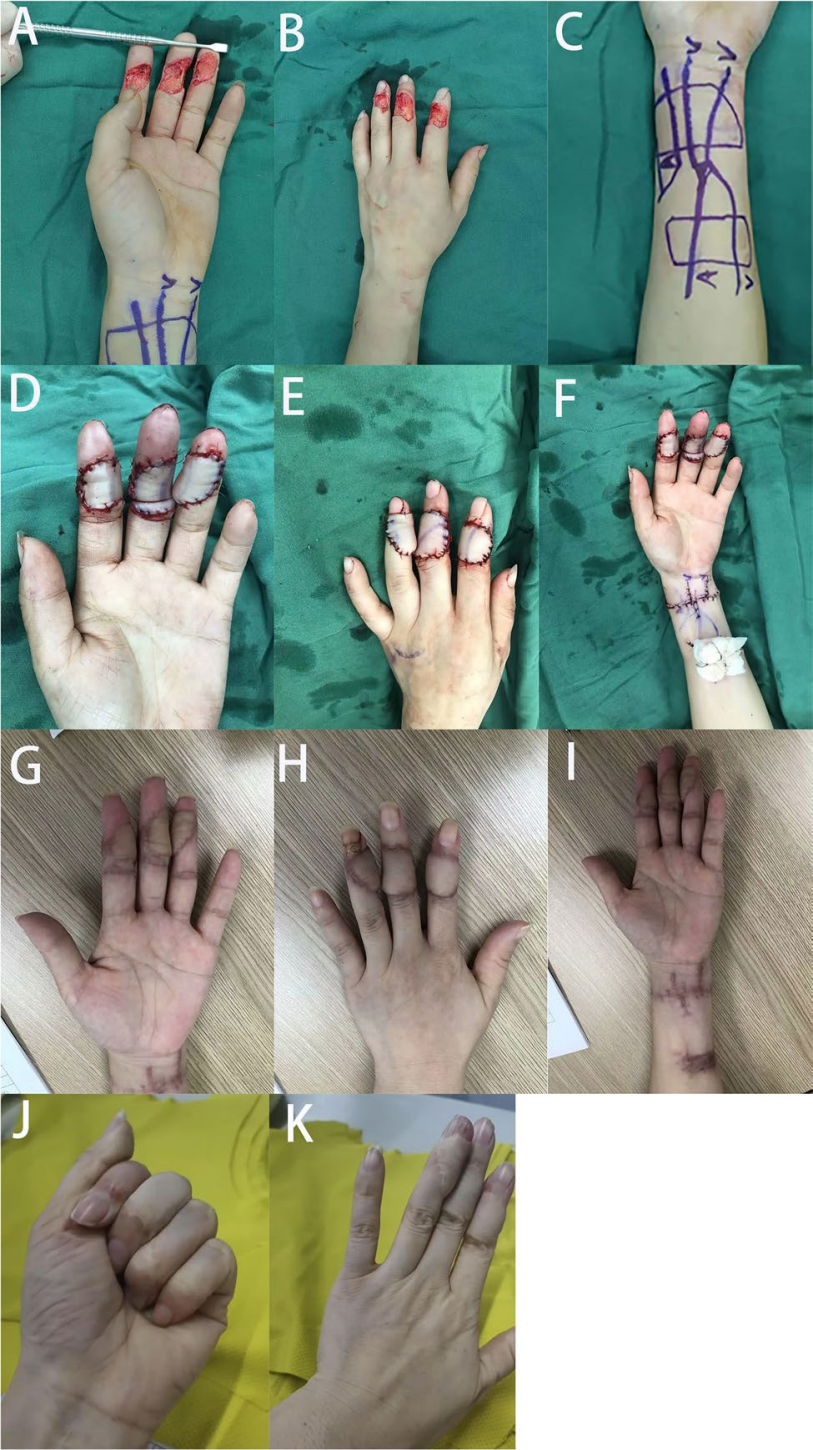
Case 2: a 32-year-old male suffered with multiple soft tissue defects and vascular injures in his left index, middle and ring fingers caused by an avulsion injury. **A**-**B** Preoperative view of the injured hand. **C** Three venous flow-through free flaps were designed from the left arm. **D**-**F** Immediate postoperative view of the flaps and the donor-site. **G**-**I** Postoperative view at follow-up. **J**-**K** Postoperative functional view

In this study, all 51 flaps survived in all patients without developing congestion or ischemia (Figs. 2 and 3). No perfusion issues was observed in the fingertip postoperatively. Table 1 presented the surgical details of the included patients, and Table 2 showed the postoperative follow-up data of the included patients. The size of defects ranged from 1.2 cm ×1.6 cm to 2.0 cm × 3.2 cm, and the size of flaps ranged from 1.5 cm ×1.8 cm to 2.7 cm × 4.0 cm. 18 of 22 patients have closed primarily at the donor sites, and four patients have skin grafts. All wounds at the donor sites healed completely. Eight cases had some degree of swelling, which resolved after conservative therapy. Blistering at the margin of the flap was observed in two fingers, and the flaps healed after dressing changes. The total complication rate was 19.6%, and no other flap congestion, infection, necrosis was observed. Three weeks after surgery, the surviving flaps displayed pink color and good capillary refill.

**Table 1:** Surgical details of the included patients

Case	Sex	Age	Injured fingers	Cause	Size of defect	Size of flap	Clinical outcomes	Donor site
1	M	32	Left (2,3,4)	Avulsion	2 × 2.5,2 × 3, 1.5 × 2.5	2 × 3, 2.5 × 3.5, 1.8 × 3	Survive	Skin graft
2	F	48	Left (3,4)	Crush	1.5 × 2, 2 × 2.5	2 × 2.5, 1.5 × 2	Survive	Closed primarily
3	M	26	Right (2,3,4)	Crush	1.2 × 1.8, 1.5 × 2,1.2 × 1.8	1.5 × 2, 2 × 2.5, 1.5 × 2	Survive	Closed primarily
4	F	36	Left (4,5)	Crush	1.5 × 2, 1.2 × 1.5	1.8 × 2.2, 1.5 × 2	Survive	Closed primarily
5	F	44	Left (2,3,4)	Crush	1.5 × 2,1.8 × 2, 1.2 × 1.5	1.8 × 2, 2 × 2.5, 1.5 × 2	Survive	Closed primarily
6	M	40	Right (4,5)	Avulsion	1.2 × 1.5, 1.2 × 1.5	1.5 × 2, 1.5 × 1.8	Survive	Closed primarily
7	M	30	Left (3,4)	Crush	1.5 × 2, 1.5 × 2.5	2 × 2.5, 2 × 3	Survive	Closed primarily
8	M	24	Right (3,4)	Avulsion	1.5 × 2.2, 2.0 × 3.2	1.8 × 3.5, 2.7 × 4.0	Survive	Closed primarily
9	F	28	Right (3,4,5)	Crush	1.5 × 2.5, 1.5 × 2.5, 1.5 × 2	2 × 3, 2 × 3, 1.8 × 2.5	Survive	Skin graft
10	M	37	Left (2,3)	Crush	1.5 × 2.5, 2 × 3	2 × 3, 2.5 × 3.2	Survive	Closed primarily
11	F	40	Right (1,3,4)	Crush	2 × 2.5, 1.5 × 2, 1.2 × 1.8	2.2 × 3, 2 × 2.5, 1.5 × 2	Survive	Skin graft
12	M	31	Right (1,2)	Avulsion	1.5 × 2, 1.5 × 1.8	2 × 2.5, 2 × 2.2	Survive	Closed primarily
13	M	22	Left (4,5)	Avulsion	1.2 × 2, 1.2 × 1.8	1.5 × 2.5, 1.5 × 2	Survive	Closed primarily
14	F	24	Right (4,5)	Crush	1.2 × 1.8, 1.5 × 1.8	1.5 × 2, 1.8 × 2	Survive	Closed primarily
15	F	38	Right (3,4)	Crush	1.5 × 2, 1.5 × 2	1.8 × 2.5, 2 × 2.5	Survive	Closed primarily
16	M	45	Left (1,2,3)	Avulsion	1.5 × 2, 1.2 × 1.8, 1.2 × 1.6	2 × 2.5, 1.5 × 2, 1.5 × 2	Survive	Skin graft
17	M	50	Right (3,4)	Crush	1.6 × 2.5, 1.6 × 2	2 × 2.8, 2 × 2.5	Survive	Closed primarily
18	M	28	Right (3,4,5)	Crush	1.2 × 1.8, 1.2 × 1.8, 1.2 × 1.6	1.5 × 2, 1.5 × 2, 1.5 × 1.8	Survive	Closed primarily
19	M	42	Left (4,5)	Avulsion	1.6 × 2.5, 1.5 × 2	2 × 3, 1.8 × 2.5	Survive	Closed primarily
20	F	35	Left (2,3)	Crush	1.8 × 2.2, 2 × 2	2 × 2.5, 2.2 × 2.5	Survive	Closed primarily
21	M	32	Right (3,4)	Crush	1.5 × 2.2, 1.5 × 2.5	2 × 2.8, 2.2 × 3.2	Survive	Closed primarily
22	M	38	Left (2,3)	Avulsion	2 × 2.2, 1.8 × 2	2.5 × 3, 2.5 × 2.8	Survive	Closed primarily

**Table 2:** Postoperative follow-up data of the included patients

Case	Complications	Additional treatment	Follow-up (month)	S-2PD Test (mm)	TAM ratio (%)	Time to return to daily function/work (month)
1	NO	No	14	Left 2: 14	85	3
				Left 3: 15	90	
				Left 4: 15	95	
2	Swelling	No	8	Left 3: 15	75	4
				Left 4: 15	80	
3	Swelling	No	10	Right 2: 16	90	5
				Right 3: 18	75	
				Right 4: 15	80	
4	NO	No	8	Left 4: 14	85	3
				Left 5: 16	90	
5	Swelling	No	16	Left 2: 14	85	4
				Left 3: 13	85	
				Left 4: 14	80	
6	NO	No	12	Right 4: 16	95	3
				Right 5: 16	90	
7	Swelling	No	10	Left 3: 15	85	4
				Left 4: 18	80	
8	NO	No	8	Right 3: 16	70	4
				Right 4: 16	70	
9	Swelling	No	8	Right 3: 16	80	3
				Right 4: 18	80	
				Right 5: 15	75	
10	Swelling	No	16	Left 2: 14	80	3
				Left 3: 16	80	
11	Swelling	No	10	Right 1: 15	75	5
				Right 3: 16	75	
				Right 4: 18	70	
12	NO	No	10	Right 1: 16	80	3
				Right 2: 16	80	
13	Blistering	Dressing changes	9	Left 4: 17	85	3
				Left 5: 15	80	
14	Swelling	No	8	Right 4: 17	90	3
				Right 5: 14	85	
15	NO	No	14	Right 3: 15	90	2
				Right 4: 16	90	
16	NO	No	14	Left 1: 16	85	4
				Left 2: 15	90	
				Left 3: 18	85	
17	NO	No	10	Right 3: 17	80	4
				Right 4: 18	80	
18	Blistering	Dressing changes	8	Right 3: 16	85	4
				Right 4: 18	80	
				Right 5: 18	80	
19	NO	No	15	Left 4: 16	75	6
				Left 5: 15	75	
20	NO	No	12	Left 2: 16	90	3
				Left 3: 16	90	
21	NO	No	11	Right 3: 15	85	4
				Right 4: 17	80	
22	NO	No	9	Left 2: 14	95	3
				Left 3: 15	90	

Mean follow-up was 10.90 ± 2.76 months (range 8 to 16 months). Satisfactory cosmetic appearance was observed in 20 cases, and the appearance was bulky in four patients. All of the flaps recovered protective sensation with the score of 2-PD 15.78 ± 1.30 mm. Using the contralateral finger as a control, the affected finger showed a mean TAM ratio of 82.84% ± 6.50%. 48 fingers were classified as good, and three fingers were classified as fair. Rate of excellent and good reached 94.12%. The average time to return to daily function or work was 3.64 ± 0.90 months.

## Discussion

To the best of our knowledge following literature review, a case series involving the use of venous flow-through free flaps for multi-finger injuries reconstruction in a single operation is not published in any current literature. In our study, the venous flow-through free flap has been successfully performed for multi-finger injuries reconstruction with a satisfactory outcome.

The extent soft tissue defect with vascular injury in multi-finger injuries has always been a challenge for reconstructive surgeons. Traditional methods for soft tissue coverage in fingers included V-Y advancement flaps, local island flaps, and digital artery island flaps. All these flaps are limited in flap size and not suitable for larger defect or multi-finger reconstruction. Conventional pedicle flaps, including abdominal and groin pedicle flaps, have been used to cover most defects in hands and multi-fingers. It was out-date with prolonged joint immobilization and multiple-stage procedures [7]. The development of microsurgery has greatly boosted the application of various free flaps for the repair of multi-finger wounds [8]. Tang et al. used multi-lobed dorsal pedis artery flaps for reconstructing complex multi-finger degloving injury [9]. However, this procedure sacrificed more soft tissue of the foot, and caused higher donor sites complications due to additional anterolateral thigh flap being transferred to cover the dorsal foot. In addition, free superficial circumflex iliac artery perforator flaps [10], free multi-lobed posterior interosseous artery perforator flaps [11], and free medial plantar artery perforator flaps [12] have been used for multi-finger defects reconstruction. A perforator flap may also be useful, but is more cumbersome than a venous flap because of the need for careful microsurgical technique to detect, preserve, and anastomose small perforators [13]. Moreover, most perforator flaps are bulky and often require defatting, especially at the flap pedicle [14]. Liu et al. compared the outcomes of finger reconstruction using arterialized venous flap, superficial palmar branch of the radial artery flap, posterior interosseous perforator flap, and ulnar artery perforator free flap. The author concluded all four types of free flaps were a practical choice in finger reconstruction for small/moderate-sized skin defects, and venous flap played an important role in such operations due to the wider indications, and better sensory recovery and cosmetic appearance associated with this method [15].

These clinical cases in our study require simultaneous vascular and soft tissue reconstruction. Flow-through flap is a good choice for such cases, as it provides an opportunity for single-stage composite reconstruction of both soft tissue and vascular defects, making it particularly useful in the reconstruction of ischemic extremities [16]. Various flow-through flaps have been reported, such as the dorsoulnar perforator flap [17], thenar flap [18], medialis pedis venous flap [14], superficial circumflex iliac artery perforator flap [19]. Among these flaps, the forearm flow-through flap is the most commonly used in clinical practice for hand injury. This flap provides a more appropriate donor in color and texture and recipient vessel size. On the volar forearm, the venous network is abundant and easy to identify with the naked eye. The flap can be harvested fast, easily, and versatile, without sacrificing any major arteries [20]. In addition, the skin of the forearm can supply flaps with sufficient size for multi-fingers defects. Harvesting of all free flaps can be performed from the single ipsilateral extremity. In our study, the size of flaps ranged from 1.5 cm ×1.8 cm to 2.7 cm × 4.0 cm. 18 of 22 patients have closed primarily at the donor sites, and four patients have skin grafts. All the donor sites healed completely.

In our cases, a flow-through vein was anastomosed to the debrided arteries at the distal and proximal of the recipient site, whereas another vein was anastomosed with the subcutaneous veins of the wound. According to the initial classification proposed by Chen et al. [21], venous flaps in our study belongs to type IV (A-V-A). The A-V-A type is mostly used for skin coverage and providing a conduit for arterial flow when the vessel is injured. There is no doubt that a viable flap depends on unobstructed arterial perfusion and adequate venous drainage. Inadequate venous drainage can lead to skin flap congestion and necrosis, while an increase in the number of draining veins can ameliorate flap venous drainage and survival [22]. In our study, blister formation occurred on two fingers, and all the flaps survived without congestion. Hughes et al. [23] reported a modification in which the flap was rotated 180 degrees prior to inset to allow unobstructed antegrade flow through the venous valves during arterialization. Liu et al. [15] suggested retrograde perfusion would enhance flap perfusion by enhancing blood flow in the periphery of venous flaps, resulting in satisfactory flap survival. In our study, we did not rotate the flap. When venous flaps are designed in the original anatomic direction (antegrade flow), the blood may bypass the peripheral tissue without perfusing the flap, which eventually leading to blister formation or partial necrosis [24]. More research is required to find the right way to achieve optimal venous drainage, reduce venous congestion and thus flap survival.

In 2022, Bashorun et al. [6] reported one case, which using multiple simultaneous venous flow-through free flap salvage for multi-finger revascularisations with an acceptable outcome. In this study, we reported our experience of more cases, which greatly enhanced the level of evidence. Our findings extended the preliminary observations by Bashorun et al. and suggested that multiple venous flow-through free flaps may be a reproducible treatment for the reconstruction of both vascular defect and soft tissue defect for complex multi-finger injuries. This progression from case report to case series represents an important step in establishing the technique’s clinical utility.

Some disadvantages should be noted. First, this study is a retrospective case series design. The absence of a control group makes it impossible to compare outcomes with alternative reconstruction methods or to establish the superiority of the described technique. More multi-center and prospective comparative design are needed in the future. Second, the venous flow through free flap requires skilled microscopic technique during surgery, with a strict monitor and nursing after the operation. Caution should be guiding microsurgeons in their early stages of advanced skill sets to minimize the risks of using such techniques. Third, for the cases with digital nerve damaged, we did not reconstruct the injured nerve. The identification and carrying over of nerves in the flap was challenging because the nerves were rather small. According to a previous study, satisfactory sensory recovery can be achieved using non-innervated flaps to repair finger defects, especially for young patients [25]. A case report by Khajuria et al. introduced a neurotized arterialized venous flow-through flap to revascularize and reconstruct a pulp defect [26]. In our study, all of the flaps recovered protective sensation with the mean score of 2-PD over 15 mm. More modifications of the flap are needed to restore sensation.

In conclusion, multiple venous flow-through free flaps can be successfully performed for the reconstruction of both vascular defect and soft tissue defect for complex multi-finger injuries in a single operation as evidenced by our study. The flap is thin, pliable, with easily identifiable vascular distribution, and no arterial sacrifice, with low donor site morbidity. Hand injuries account for a significant proportion of emergency cases and impose a heavy socio-economic burden. The high health-care and productivity costs highlight the urgency of strengthening research on hand and multi-finger injuries. Listing such injuries as a key area for trauma research and clinical treatment will help formulate targeted intervention strategies, thereby reducing the overall burden they impose on the medical system and society.

## Data Availability

All data generated or analysed during this study are included in this published article.
